# Application of Ferulic Acid for Alzheimer’s Disease: Combination of Text Mining and Experimental Validation

**DOI:** 10.3389/fninf.2018.00031

**Published:** 2018-05-29

**Authors:** Guilin Meng, Xiulin Meng, Xiaoye Ma, Gengping Zhang, Xiaolin Hu, Aiping Jin, Yanxin Zhao, Xueyuan Liu

**Affiliations:** ^1^Shanghai Tenth People’s Hospital, Tongji University School of Medicine, Shanghai, China; ^2^School of Computer Science and Informatics, Indiana University, Bloomington, IN, United States; ^3^Houma People’s Hospital, Linfen, China; ^4^Library of Tongji University, Shanghai, China; ^5^School of Life Sciences, Tsinghua University, Beijing, China

**Keywords:** Alzheimer disease, BACE1, curcumin, ferulic acid, MMP2, STRING, text mining

## Abstract

Alzheimer’s disease (AD) is an increasing concern in human health. Despite significant research, highly effective drugs to treat AD are lacking. The present study describes the text mining process to identify drug candidates from a traditional Chinese medicine (TCM) database, along with associated protein target mechanisms. We carried out text mining to identify literatures that referenced both AD and TCM and focused on identifying compounds and protein targets of interest. After targeting one potential TCM candidate, corresponding protein-protein interaction (PPI) networks were assembled in STRING to decipher the most possible mechanism of action. This was followed by validation using Western blot and co-immunoprecipitation in an AD cell model. The text mining strategy using a vast amount of AD-related literature and the TCM database identified curcumin, whose major component was ferulic acid (FA). This was used as a key candidate compound for further study. Using the top calculated interaction score in STRING, BACE1 and MMP2 were implicated in the activity of FA in AD. Exposure of SHSY5Y-APP cells to FA resulted in the decrease in expression levels of BACE-1 and APP, while the expression of MMP-2 and MMP-9 increased in a dose-dependent manner. This suggests that FA induced BACE1 and MMP2 pathways maybe novel potential mechanisms involved in AD. The text mining of literature and TCM database related to AD suggested FA as a promising TCM ingredient for the treatment of AD. Potential mechanisms interconnected and integrated with Aβ aggregation inhibition and extracellular matrix remodeling underlying the activity of FA were identified using *in vitro* studies.

## Introduction

Alzheimer’s disease (AD) is a chronic neurodegenerative disease that usually progresses from short memory loss to dementia, and accounts for 50%–70% of dementia cases (Burns and Iliffe, 2009). According to the World Alzheimer Report (Prince, 2015), 46.8 million people worldwide are living with dementia, and this number is estimated to reach 131.5 million by 2050, which will result in an increasing burden on society and families. In addition, the cost of long-term care, home services, and non-professional caregivers is greater than the cost of direct medical care (Bullock, 2004; Winblad et al., 2016; Yokoyama et al., 2016).

Despite enormous financial and research investments, appropriate interventions to prevent the progress of AD are lacking (Iqbal and Grundke-Iqbal, 2011; Selkoe, 2013). Based on the failure of a number of novel AD drugs, investigators are increasingly convinced that AD is not a single but rather a multifactorial disease (Iqbal et al., 2013), and hence, drugs that target one node on the classical pathway have little effect on the AD disease network. Since AD is a multifactorial disease, drugs that modulate systemic or multiple targets are of interest.

Traditional Chinese medicine (TCM) compositions usually exert systemic impact and can be a source of drug repositioning efforts (Wang et al., 2011). TCM treatments are natural herbs discovered by the ancient Chinese and evolved through at least 3000 years of clinical practice. TCM is gaining increasing attention with the emergence of integrative and personalized medicine, characterized by pattern differentiation on individual variance and treatments based on natural herbal synergism (Wang and Wei, 2009). With the growing popularity and promising approach of TCM applicability, the ever-increasing demand for understanding the pharmacological mechanisms and potential drug efficacy are the major issues that need to be addressed.

In this study, we sought to shed light on TCM for AD. What typical TCM treatments could be effective for AD, and what are the underlying target-based mechanisms? How can we integrate systemic and target-based understandings of the disease and treatments (Cho et al., 2006)? With the overwhelming amount of biomedical knowledge recorded in texts, text mining is essential for identifying, extracting, managing, integrating and exploiting this information to discover new, hidden, or unsuspected information. Text mining is a computer-based discovery of new, previously unknown information, which automatically extracts information from different written resources (Ding et al., 2013), drawing on information retrieval, statistics, and computational linguistics. It has considerable potential for drug target discovery and re-labeling of existing drugs. Some typical proven drug repositioning cases are available for text mining, such as the beneficial effect of estrogen on human memory discovered by Smalheiser and Swanson (1996), thalidomide for treating acute pancreatitis extracted by Weeber et al. (2003), and the association of migraine with AMPA receptors identified using Litlinker (Yetisgen-Yildiz and Pratt, 2006).

Herein, we report an approach for finding an appropriate TCM for AD through the utilization of text-mining from literature database, exploring the underlying therapeutic mechanisms followed by searching for protein-protein interactions (PPI) using the STRING platform, and finally using the SHSY5Y-APP AD cell line model for validation.

## Materials and Methods

**Figure 1:**
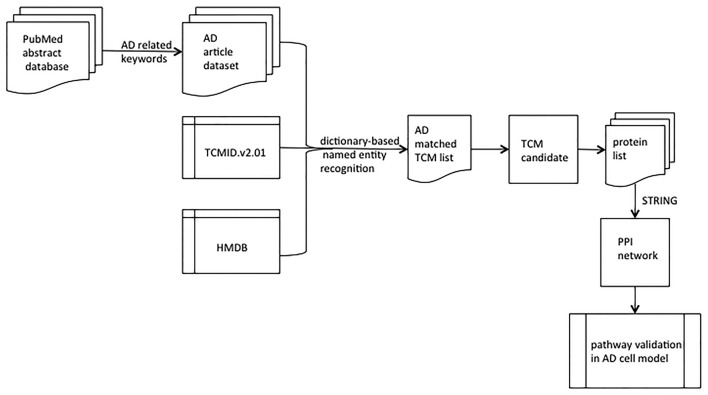
Flowchart for selecting TCM candidates for validation. AD, Alzheimer’s disease; TCM, traditional Chinese medicine; TCMID.v2.01, a TCM database; HMDB, human metabolomics database; STRING, a platform of protein-protein interaction (PPI).

Our first aim was to select a TCM candidate from the extensive literature collection. The study workflow is shown in Figure 1.

### Data Collection and Extraction to Find a TCM Candidate for AD

First, we assembled an AD literature dataset by retrieving articles from PubMed using AD-related keywords: “Alzheimer or Mild cognitive impairment or Dementia or Significant memory concern or Subjective memory complaint,” a resource for extracting and defining TCM candidates. The TCM database, TCMID.v2.01 (Chen et al., 2006), was then utilized, which included names, stitch_id, PubChem_id, synonyms, formula, SMILES strings, and the source of the involved chemicals. The TCM terminologies, mentioned in the abstracts of the retrieved articles were then extracted using dictionary-based named entity recognition (i.e., simple word matching) provided by LingPipe^1^. Using this method, the selection of the TCM for AD was retrieved in a relatively short duration.

Furthermore, we matched the PubChem ID of TCM and the origin ontology in the human metabolome database (HMDB; Southan et al., 2013) to determine the optimal TCM candidates. After focusing on a possible lead TCM candidate, we retrieved articles in the AD dataset, and extracted protein names that co-occurred with the candidate TCM using the dictionary-based entity recognition from NCBI protein list^2^. This gave us with a list of possible proteins affected by the TCM candidate.

### Inferring Possible PPI (Li et al., 2017) Networks of the TCM Candidate Using STRING

STRING presents a specific and productive functional relationship between two proteins into a combined interaction confidence score, which is derived from the co-expression score, experimentally determined interaction score, and the automated text mining score. In this system, the automated text mining score is higher than or approximately equal to the experimentally determined interaction score since it is integrated from these scores. However, if the text mining score was lower than the experimentally determined interaction, there were two possibilities: (1) the experimentally determined interaction score was a false positive; (2) only a few studies are available related to these two proteins; however, experimental validation could be conducted.

We deposited the protein list mentioned above in the multiple protein column in the search webpage of STRING^3^ and acquired the PPI network after deleting results with co-expression scores >0 (already validated PPI), or experimentally determined interaction score × automated text mining score = 0 (little relevance). In the next step, we rearranged the PPI network according to text mining scores and obtained the top proteins in the network, which were most likely related to candidate mechanisms in AD.

### Validation of Protein Expression and PPI in an AD Cell Line Model

In order to generate strong evidence not only in the data level, we validated the possible mechanisms mined from STRING using AD cell lines.

### Cell Culture and Treatment With TCM Candidate for AD

SHSY5Y-APP cells, a classic cell line for AD research, were a kind gift from Shanghai Jiao Tong University. The cells were cultured in MEM supplemented with 10% heat-inactivated fetal bovine serum (FBS), 100 units/mL penicillin, and 100 μg/mL streptomycin (Invitrogen, Carlsbad, CA, USA) at 95% humidity, 37°C, and 5% CO_2_ in an incubator. The cells were passaged by trypsinization every 2–3 days. The SHSY5Y-APP cells were treated with different doses of the TCM candidate for 24 h. In the existing researches, the effective ferulic acid (FA) concentrations vary from 10 nM to 1 mM without toxic reactions in a variety of cell lines, in accordance with the point that FA is highly safe for daily and long-term consumption (Thakkar et al., 2015; Sompong et al., 2017; Zhang et al., 2017). In line with a previous study using the same cell line (Cui et al., 2013), micromolar (μM) was chosen as the unit for FA concentration and upgraded in steps of 0 μM, 15 μM, 30 μM and 60 μM.

### Co-immunoprecipitation Assays (co-IP)

Cell lysates were centrifuged (10,000× *g*) at 4°C for 15 min. Proteins were then immunoprecipitated with the relevant antibodies to determine interactions. The precleared Protein A/G Plus-Agarose beads (Merck KGaA, Darmstadt, Germany) were incubated with the immunocomplexes for 2 h and washed four times with phosphate-buffered saline. The immunoprecipitates were subjected to sodium dodecyl sulfate-polyacrylamide gel electrophoresis (SDS-PAGE; Merck KGaA, Darmstadt, Germany), followed by transfer to polyvinylidene difluoride (PVDF) membrane (Amresco, OH, USA). The antibody-antigen complexes were visualized using the UPV software according to the manufacturer’s instructions. The immunoreactive bands were quantified to confirm the appropriate levels of proteins.

### Western Blot

#### Preparation of Protein Samples

After TCM candidate exposure for 24 h, SHSY5Y-APP cells were washed with pre-cooled 4°C PBS, and then the wash solution was discarded. The above procedure was repeated twice. PMSF was added to lyse the cells on ice with frequent shaking for 30 min. After lysis, the cells were scraped with a clean scraper, and then the cell debris and lysate were transferred and centrifuged at 12,000 rpm for 5 min at 4°C. The supernatant after centrifugation was stored at −20°C.

#### Determination of Protein Concentration

The standard BCA assay procedure was done as previously described (Huang et al., 2010). After blocking, the membranes were probed with the following primary antibodies (Cell Signaling, Beverly, MA, USA) using different dilutions: rabbit anti-MMP2 (92 kDa, Abcam ab92539, 1:2000), rabbit anti-MMP9 (92 kDa, Abcam ab38898, 1:2000), rabbit anti-BACE1 (68 kDa, Abcam ab183612, 1:1000), rabbit anti-APP (87 kDa, Abcam ab15272, 1:600), and mouse anti-beta-actin (42 kDa, Boster, BM0627, 1:200). All experiments were performed at least three times.

#### Electrophoresis

We prepared the 12% separation gel, 10% separation gel and 5% concentration gel. The prepared protein sample and the maker were added to 40 μg. After the sample was added, constant 80 V electrophoresis was performed until the bromophenol blue indicator was linear at the junction of the concentrated gel and the separation gel, and the pressure was changed to constant 120 V. This process took about 1.5 h. Next, we removed the gel and the target band according to the Marker. The PVDF membrane was soaked in methanol for several seconds and soaked in the electroporation buffer together with the filter paper. The transfer membrane conditions were as below: β-actin 200 mA 90 min, BACE1 200 mA 120 min, APP, MMP2 and MMP9 250 mA 120 min.

#### Immunoblotting and Analysis

The PVDF membrane was soaked in TBST containing 5% skimmed milk powder and shaken at room temperature for 2 h. We mixed the ECL reagent with the stable peroxidase solution in a ratio of 1:1, added the solution onto the PVDF membrane. X-ray film was placed in the solution, flushed, dried, scanned, and finally analyzed grayscale value with BandScan 5.0 software (NIH, USA). Statistical analysis was performed using SPSS 20.0 software (SPSS, Chicago, IL, USA). Quantitative data are presented as mean ± standard deviation (SD) of triplicates in an independent experiment that was repeated three times. Data were compared using Student’s unpaired *t*-test for direct comparison between two-groups and the Tukey-Kramer test after a significant one-way analysis of variance (ANOVA), and *F-test* for multiple-group comparisons. *P* < 0.05 was considered as statistically significant.

## Results

### Text Mining Using AD Literature and the TCM Database

We retrieved 195,882 articles from PubMed using AD-related keywords and assembled an AD article dataset.

After matching TCMID.v2.01 ingredients to the AD article database, we extracted a list of AD-related TCM ingredients with PubChem IDs, which was checked for origin ontology in HMDB.

We ranked the TCM ingredients by the number of mentions and focused on the top 20 frequent terms after deleting common words, such as “protein,” “glucose,” “amino acid,” and others (Table 1).

**Table 1:** TCM ingredients’ AD-matched list in PubMed.

No.	Frequency	TCM ingredients	PubChem ID	Origin ontology in HMDB
1	41288	Tau	156615	Endogenous
2	5431	Glutamate	5128032	Bacteria and beans
3	3609	Cholinesterase	4460501	Not available
4	3557	Acetylcholine	5315629	Endogenous
5	3453	Dopamine	681	Endogenous
6	2824	Choline	305	Many plants and animal organs
7	1999	Nicotine	89594	Tobacco
8	1973	Melatonin	896	Endogenous
9	1898	Glutathione	124886	Drug metabolite and Endogenous
10	1722	Aspartate	5460541	Endogenous
11	1718	Serotonin	5202	Endogenous
12	1593	Tyrosine	6057	Endogenous
13	1534	Scopolamine	5184	Solanaceae
14	1432	Serine	5951	Endogenous
15	1372	*Curcumin*	969516	*Curcuma longa*
16	1089	Levodopa	6047	Endogenous
17	939	Estradiol	5757	Drug, Endogenous, Food
18	931	Methionine	6137	Drug metabolite and Food
19	788	Creatine	586	Endogenous
20	785	Physostigmine	5983	Drug

*TCM, traditional Chinese medicine; HMDB, human metabolome database*.

Next, we checked the origin ontology of all the 20 components. In Table 1, a total of 12 endogenous ingredients, including Tau, Acetylcholine, Dopamine, Melatonin, Glutathione, Aspartate, Serotonin, Tyrosine, Serine, Levodopa, Estradiol and Creatine were selected. These endogenous ingredients could not only be absorbed from the environment but also produced and synthesized within the organism or system. Cholinesterase does not have an origin result in HMDB, and hence, our list was narrowed down to Glutamate, Choline, Nicotine, Scopolamine, Curcumin, Methionine and Physostigmine, of which Glutamate, Choline, Methionine and Physostigmine could be extracted from a broad list of drug and food options, in which includes Nicotine, Scopolamine and Curcumin. Literature suggested that Scopolamine induced retrograde amnesia, or an inability to recall events prior to its administration (Colettis et al., 2014), and hence, it was deleted from our list. Compared to the double-edged function of Nicotine, Curcumin has not been shown to cause any toxicity despite its daily consumption for centuries in Asian countries (Maheshwari et al., 2006). Thus, we first focused on Curcumin in the candidate list, an obvious TCM component that is extracted from Curcuma longa, a common plant in China.

In order to confirm its effect, we extracted all the sentences that contained “curcumin” from the AD article database. From the 107 retrieved sentences, one sentence inferred that Curcumin was suitable for treating AD; whereas, FA appeared in the same sentence with Curcumin at a high frequency. Three representative sentences are shown in Table 2.

**Table 2:** Sentences retrieved from the literature based on the entities’ biomedical researches.

PMID	Sentences retrieved from literature
16387689	Because it can modulate the expression of these targets, curcumin is now being used to treat cancer, arthritis, diabetes, Crohn’s disease, cardiovascular diseases, osteoporosis, Alzheimer’s disease, psoriasis and other pathologies.
17127365	Food supplementation with curcumin and ferulic acid is considered a nutritional approach to reduce oxidative damage and amyloid pathology in Alzheimer’s disease.
26592858	Ferulic acid has structural similarity with curcumin, which is known for its monoamine oxidase (MAO) inhibitory activity.

Curcumin and FA share some similarities, and as a major metabolite of curcumin, FA has better bioavailability and metabolic stability than curcumin, thus rendering it as a better candidate (Badavath et al., 2016). Thus, we re-assigned our TCM target from curcumin to FA. FA also denoted as 3-(4-hydroxy- 3-methoxyphenyl)-2-propenoic acid, has the following chemical structure:


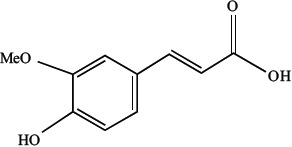


After selecting FA as our TCM target, the next step was to understand the possible mechanism of FA in AD. Given the apparent complexity of the FA mechanism network, understanding its involvement in the underlying AD pathological pathways was a challenge.

We retrieved 178,725 articles using FA-related keywords “Curcumin, or FA, or Sodium Ferulate” in PubMed. From these articles, we extracted a list of proteins that was co-mentioned with FA, using the dictionary-based entity recognition. This resulted in 178 proteins that were ranked by the number of times mentioned with links to sentence sources in PubMed. After deleting a large number of false positives using the auto stop list (Fenner, 2008) of drug abbreviations, experimental test abbreviations, cell lines, synonyms of other genes, and common serum proteins, we reduced the list of proteins to 20, which are listed below:

APOB, BACE1, BCL2, CCNB1, CCND1, ERBB2, GAPDH, GSR, HMOX1, MMP2, MYB, NOS1, PCNA, PEA15, PIK3CA, PPARA, PTGS2, RAF1, TXN and VEGFA.

### Potential PPI Network in STRING

**Figure 2:**
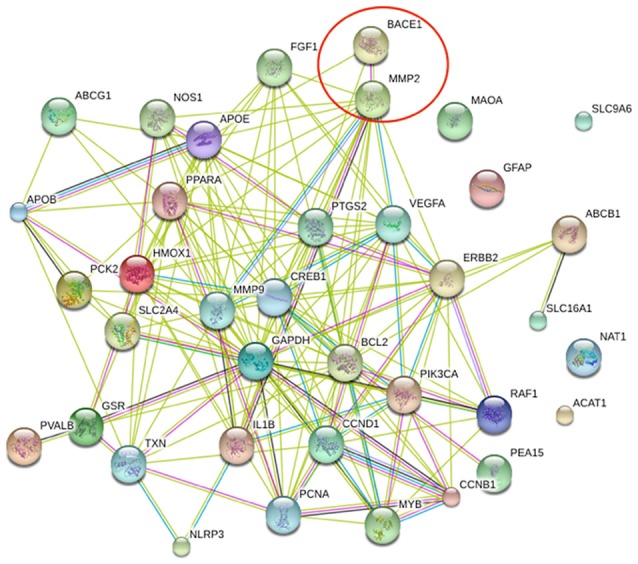
PP1 map generated by STRING showing the interactions of the selected 20 proteins Edges represent protein-protein associations, 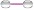 interactions from experimentally determined, 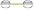 text mining, 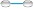 known interactions from curated databases, 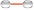 gene fusions, 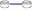 gene co-occurrence, 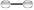 co-expression and 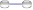 protein homology.

Next, we entered these proteins into STRING in order to obtain direct as well as indirect protein associations (Figure 2).

The PPI scores were also exported into Table 3. The BACE1 and MMP2 combined score ranked on top among the interactions, however it had a lower automated text mining score than in the experimentally determined interaction score. We selected BACE1-MMP2 interaction as the target PPI. The edges connecting BACE1 and MMP2 (Figure 1) are 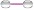 and 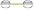, which indicates that BACE1 and MMP2 may interact with each other. However, when we searched for *in-silico* evidence, the two words occurred in the full-text of some experimental articles, albeit without any direct correlation, such that the experimentally determined interaction was a false positive with a high validation possibility.

**Table 3:** Ranking scores of protein interactions in STRING.

Node 1	Node 2	Co-expression	Experimentally determined interaction	Automated text mining	Combined score
BACE1	MMP2	0	0.534	0.149	0.586
PPARA	APOB	0.053	0	0.578	0.583
CCND1	MMP2	0	0	0.575	0.575
NOS1	BCL2	0.048	0	0.566	0.569
APOB	GAPDH	0	0.179	0.491	0.564
BCL2	PPARA	0	0.377	0.304	0.548
MYB	GAPDH	0.061	0	0.517	0.527
RAF1	GAPDH	0.059	0	0.503	0.512
TXN	BCL2	0	0	0.507	0.507
PCNA	PTGS2	0	0	0.507	0.507
PCNA	TXN	0.2	0.102	0.367	0.506
PCNA	MMP2	0	0	0.506	0.506
PPARA	GAPDH	0	0.042	0.501	0.501
NOS1	GAPDH	0.084	0	0.476	0.5
ERBB2	RAF1	0	0.292	0.76	0.487
BACE1	GAPDH	0.053	0	0.465	0.472
ERBB2	CCNB1	0	0	0.454	0.454
BCL2	GSR	0.048	0	0.446	0.45
NOS1	MMP2	0.055	0	0.442	0.45
TXN	VEGFA	0	0	0.442	0.442
PCNA	MYB	0.058	0	0.426	0.436
VEGFA	CCNB1	0	0	0.432	0.432
TXN	APOB	0	0	0.417	0.416
MYB	PIK3CA	0.055	0.104	0.349	0.401

### Novel Hypothesis for the FA Related Mechanism in AD

Based on the above results, we hypothesized that BACE1 and MMP2 were closely linked to the mechanism of FA. The two possibilities are as follows: these two proteins interacted directly, which could be validated by co-IP; in addition to proteolytic cleaving of the amyloid precursor protein (APP), the extracellular matrix proteins may also have a role in the AD pathological pathways, and these two pathways were always concurrent in AD.

**Figure 3:**
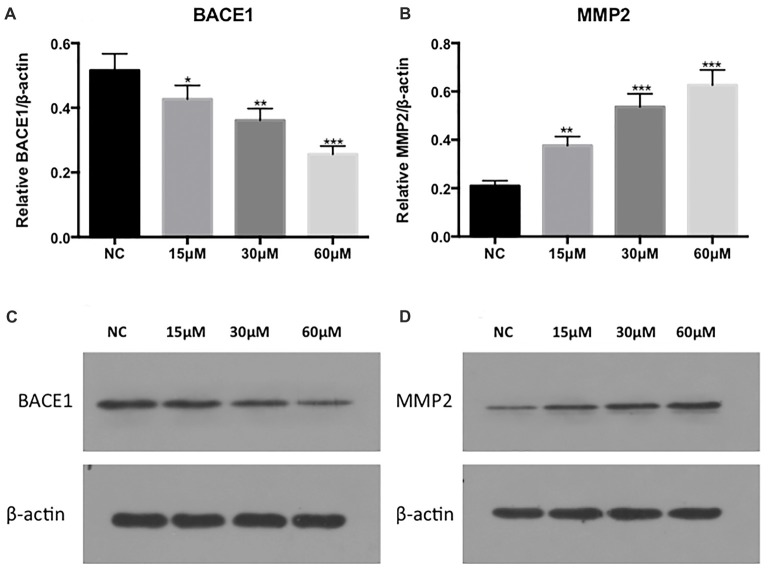
Protein expression of BACE-1 and MMP-2 after exposure to 0, 15, 30 and 60 μM ferulic acid (FA). **(A,B)** are the expression analysis results of **(C,D)**. Statistical significance is denoted by *p < 0.05, **p < 0.01, ***p < 0.001 (one-way ANOVA; N = NC).

The SHSY5Y-APP cell lines were passaged every 2–3 days by trypsinization, and treated with 0, 15, 30 and 60 μM FA (Yuanmu Tech, China) for 24 h. After FA exposure for 24 h, the BACE-1 expression decreased and MMP-2 expression increased in a dose-dependent manner (Figure 3).

**Figure 4:**
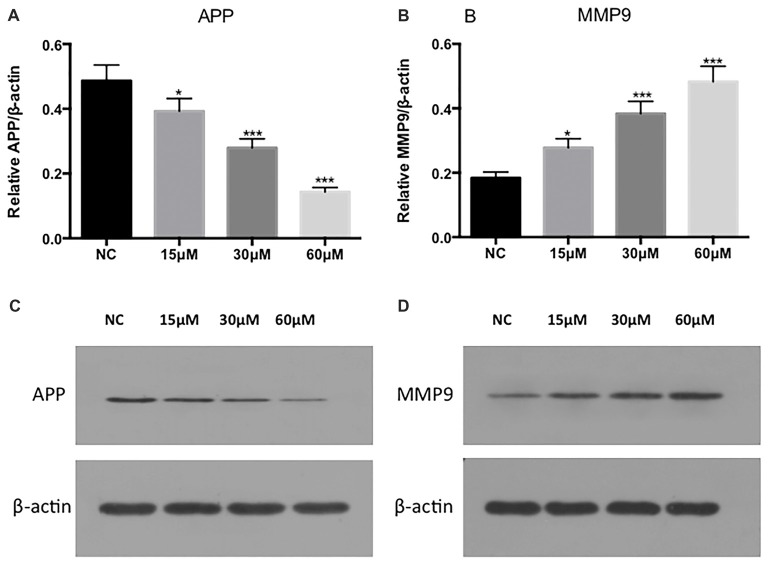
Protein expression of APP and MMP9 after exposure to 0, 15, 30 and 60 μM FA. **(A,B)** are the expression analysis results of **(C,D)**. Statistical significance is denoted by *p < 0.05, ***p < 0.001 (one-way ANOVA; N = NC).

We next tested the expression of APP, another dominant protein in the Aβ aggregation pathway, which is positively correlated with BACE1. After exposure to FA, the proteolytic cleavage of APP and APP enzymolysis is decreased, thereby improving the AD process. In addition, we tested MMP9, another protein in the extracellular matrix pathway. We observed that extracellular matrix protein expression was increased after FA exposure and contributes to the pathological process of AD (Figure 4).

## Discussion

### TCM Candidate Selection for AD Using Data Mining

In this study, we used text mining to select a TCM candidate for subsequent validation. FA was selected and *in vitro* validation was performed to understand the potential mechanisms involved in AD. Furthermore, the current study used the information-medicine integrated system to map TCM for AD research. In addition, text mining was coupled to the experimental validation to assess the drug selection outcomes. This offset the information gap and maximized the utilization of existing knowledge to select the optimal TCM candidate to study.

Drug discovery for AD is no longer a game of chance or just limited to the availability of new technology. Societal expectations about drug efficacy are rising; thus, early-stage drug discovery necessitates accessible, standardized data sets to generate a complete scenario of the physiological function and disease relevance. Some pioneering studies have focused on drug repurposing, such as systematic “omics” data mining of genome-wide association studies (GWAS), HMDB, epigenomics and proteomics data (Zhang et al., 2016; Pimplikar, 2017). These studies suggested drugs that were applicable for other diseases having novel anti-AD indications. These attempts were very logical however these studies did not consider TCM as a source of information for AD research. TCM is a good source for drug discovery. The uniqueness of the TCM system is based on the philosophical logic underlying daily practices (Ho et al., 2011), which was accumulated over thousands of years of empirical studies and provides a unique view of the relationships between the human body and the universe (Gu and Chen, 2014). Therefore, a better understanding of TCM and key learning from the past with appropriate strategies for the future is essential to make a significant difference. These theories render the proposed approach useful in identifying novel relationships between diseases and drugs that have a high probability of being physiologically effective. On the other hand, existing TCM drug mining primarily focuses on the assessment of ancient classic literature, with less analysis of herbal components (May et al., 2014; Pae et al., 2016), and thus may affect knowledge dissemination. Our study is the first to combine well-known TCM database with text mining approaches. This led us to select FA as a lead candidate for experimental validation for AD.

### MMP2-BACE1 Mechanisms of FA in AD

Our findings suggested that FA might be a promising multi-targeted TCM with a therapeutic potential for AD (Jung et al., 2016). We evaluated the APP and BACE1 inhibitory activities, which inhibit Ab aggregation; in addition, the matrix clearance properties of MMP-2 and MMP-9 implicated FA was actively involved in the alteration of matrix proteins and that it played a major role in *in vitro* extracellular matrix remodeling. As shown in a previous *in situ* proximity ligation assay (*in situ* PLA), which is a new technique to monitor PPI with high specificity and sensitivity, it was found that APP, MMP2 and MMP9 all interacted with TGFB1, and the interaction of MMP2 and BACE1 was also positive (Chen et al., 2014). Furthermore, from the Human Protein Reference Database (HPRD) in the STRING platform (Higashi and Miyazaki, 2003), the COOH-terminal parts of APP were found to interact with the extracellular matrix and highly selectively inhibit MMP2, in which the decapeptide region of APP was likely an active site-directed inhibitor toward MMP2.

The pathways of PPI at the molecular level include cellular transduction and biological function. Hence, the two pathways of Aβ aggregation inhibition and extracellular matrix remodeling were interconnected and integrated to the biological function-signaling map for AD. The results of these analyses might have potential application in exploring FA mechanism because they can be used as rational targets to inhibit the function of pathways essential to AD. In the multi-targeted AD model, APP cleavage, inhibition of Aβ deposition, and extracellular matrix remodeling are co-operative interactions involved in AD pathology, which could be attractive therapeutics with respect to pharmacokinetics and pharmacodynamics when compared to a specific highly specific single target molecule. These results highlight the prospective beneficial effects of FA as a therapeutic agent against AD pathology.

One limitation of this study was that no animal model was validated, and the experimental validation in the AD cell model was not sufficient to make a conclusive statement regarding the potential efficacy and benefit of FA. However, we found evidence in previous study of FA’s protective effects on different animal models of intra-cerebroventricular (i.c.v.) injection of Aß1–42 in mice and APP/PS1 mutant transgenic mice (Jung et al., 2016), which shows potent anti-oxidant and anti-inflammatory activities. Future studies should discuss the in-depth mechanisms of FA together with the physiological data to evaluate FA efficacy and involvement in an AD animal model. These include: What are the safety implications of the different doses of FA for AD? What biomarkers exist for FA metabolites? In addition, understanding the role of FA within the system, the pathways and networks of the different protein interactions are invaluable.

## Conclusion

In summary, we demonstrate that the combination of text mining and professional medical knowledge is an effective approach for finding new mechanisms underlying the clinical therapeutics for AD. Equipped with this data, the clinical scientist can obtain information in a short period of time without searching large volumes of articles. Moreover, using *in vitro* studies for validation, the data-driven results were based on not only a hypothesis but also true novel findings of potential mechanisms interconnected and integrated by Aβ aggregation inhibition and extracellular matrix remodeling underlying the activity of FA. The present study strongly supported text mining of the ever-increasing volume of literature and TCM database as a drug repositioning approach for elucidating FA as a promising TCM ingredient for treating AD.

## Author Contributions

GM, YZ and XMeng designed the study. XMa and AJ performed experiments and prepared figures. XMa, GZ and XH analyzed the data. GM and XMeng wrote and discussed all sections of the manuscript. All authors reviewed and approved the manuscript.

## Conflict of Interest Statement

The authors declare that the research was conducted in the absence of any commercial or financial relationships that could be construed as a potential conflict of interest.

## Footnotes

^1^http://alias-i.com/lingpipe/index.html

^2^https://www.ncbi.nlm.nih.gov/protein/

^3^https://string-db.org/
